# 

**Published:** 1857-11

**Authors:** 


NORTH AMERICAN

### MEDICO-CHIRURGICAL REVIEW.

NO. 6.—NOVEMBER, 1857.

### CONTENTS.

### ANALYTICAL AND CRITICAL REVIEWS.

PAGE

ART. I.—De l'Influence de la Navigation et des Pays Chauds, sur la Marche de la Phthisie Pulmonaire. Par M. JULES ROCHARD, . . . 801
On the Effects of Climate on Tuberculous Disease. By EDWARD LEE, M.R.C.S., . . . . . . . . . . . . . 801
Le Climat de l'Italie sous le Rapport Hygienique et Medical. Par le DOCTEUR ED. CARRIERE, . . . . . . . . . 801
On the Curative Influence of the Climate of Pau, and of the Mineral Waters of the Pyrenees in Diseases. By M. A. TAYLOR, M.D., . . . . . . . . . . . . . . . 801

ART. II.—Lehrbuch der Krankheiten der Weiblichen Sexualorgane. Von DR. F. W. SCANZONI, . . . . . . . . . . 832
Text-Book of Diseases of the Female Sexual Organs. By DR. F. W. SCANZONI, . . . . . . . . . . . 832

ART. III.—Clinical Lectures on Certain Diseases of the Urinary Organs. By ROBT. B. TODD, M.D., &c., . . . . . . . . 858

ART. IV.—Memorias sobre la Topografia Medica de la Habana. Par el DOCT. DON MARCIAL DUPIERRIS, . . . . . . . . 870
Medical Topography of Havana. By DOCTOR DUPIERRIS, . 870

ART. V.—Climatology of the United States, &c. By LORIN BLODGETT, . 874

ART. VI.—Principles of Medicine. By C. J. B. WILLIAMS, M.D., &c., . 877

ART. VII.—Diseases of the Ear. By J. NOTTINGHAM, . . . . . 878

ART. VIII.—On Diseases of the Skin. By E. WILSON, F.R.S., &c., . . 879

ART. IX.—Catalogue of the Medical Library of Pennsylvania Hospital. By E. FISCHER, M.D., . . . . . . . . . . 880

ART. X.—The Practice of Surgery. By JAS. MILLER, F.R.S.E, &c.,. . 882

ART. XI.—Address Before the Pennsylvania State Medical Society. By R. LA ROCHE, M.D., . . . . . . . . . . 883

PAGE
ART. XII.—A Treatise on Midwifery, &c. By P. CAZEAU, . . . . 883
ART. XIII.—Physician's Visiting List, Diary, &c., . . . . . . 884
ART. XIV.—Materia Medica and Therapeutics. By T. D. MITCHELL, M.D., &c., . . . . . . . . . . . . 884

### ORIGINAL COMMUNICATIONS.

ART. I.—Urethro-Vaginal and Vesico-Vaginal Fistules. By N. BOZEMAN, M.D., . . . . . . . . . . . . . . . 885
ART. II.—On the Diversity of Symptoms in Scarlatina Maligna. By G. SUTTON, M.D., . . . . . . . . . . . . . 912
ART. III.—Psychology of Acute Rheumatism. By A. T. WHEELOCK, M.D., &c., . . . . . . . . . . . . . 922
ART. IV.—On the Treatment of Prolapsus Uteri by the Local Application of Tannin. By G. A. KUNKLER, M.D., . . . . . . . 927
ART. V.—Encephaloid Tumor within the Cranium and on the Neck. By J. C. KLUGE, M.D., . . . . . . . . . . . . 929
ART. VI.—Puerperal Fever and Erysipelas. By R. V. DUNCAN, M.D., . 931
ART. VII.—Dislocation of the Femur into the Sacro-Ischiatic Notch, reduced by Manipulation. By G. R. HENRY, M.D., . . . . 933
ART. VIII.—Mortality of Philadelphia, for April, May, and June, 1857. By W. JEWELL, M.D., . . . . . . . . . . 934

### EDITORS' TABLE.

Female Medical Colleges and Female Doctors, . . . . . . . . 942
Medical Teaching and Opportunities in Philadelphia, . . . . . 948
Miscellaneous, . . . . . . . . . . . . . . . . . 949

### NECROLOGICAL.

Dr. Marshall Hall, . . . . . . . . . . . . . . . 952

